# English Pig Farmers’ Knowledge and Behaviour towards African Swine Fever Suspicion and Reporting

**DOI:** 10.1371/journal.pone.0161431

**Published:** 2016-09-29

**Authors:** Claire Guinat, Ben Wall, Linda Dixon, Dirk Udo Pfeiffer

**Affiliations:** 1 Veterinary Epidemiology, Economics and Public Health Group, Royal Veterinary College, Hawkshead Lane, Hatfield, Hertfordshire, AL9 7TA, United Kingdom; 2 The Pirbright Institute, Pirbright laboratory, Ash road, Pirbright, Surrey, GU24 0NF, United Kingdom; Universidade Federal de Pelotas, BRAZIL

## Abstract

African swine fever (ASF) is a notifiable, virulent swine disease, and is a major threat to animal health and trade for many European Union (EU) countries. Early detection of the introduction of ASF virus is of paramount importance to be able to limit the potential extent of outbreaks. However, the timely and accurate reporting of ASF primary cases strongly depends on how familiar pig farmers are with the clinical signs, and their motivation to report the disease. Here, an online questionnaire survey was conducted between December 2014 and April 2015 to investigate English pig farmers’ knowledge and behaviour towards ASF in terms of clinical suspicion and reporting. Multivariable logistic regression analysis was used to identify factors influencing the two variables of interest: 1) farmers who “would immediately suspect ASF” if they observed clinical signs of fever, lethargy, reduced eating and high mortality on their farm and 2) farmers who “would immediately report ASF” if they suspected ASF on their farm. The questionnaire was completed by 109 pig farmers. Results indicate that pig farmers having poor knowledge about ASF clinical signs and limited concern about ASF compared with other pig diseases are less likely to consider the possibility of an outbreak of ASF on their farm. In addition, pig farmers lacking awareness of outbreaks in other countries, having a perception of the negative impact on them resulting from false positive reporting and the perceived complexity of reporting procedures are less likely to report an ASF suspicion. These findings indicate important areas for educational campaigns targeted at English pig farmers to focus on in an attempt to increase the likelihood of a rapid response in the event of an ASF outbreak.

## Introduction

The circulation of African swine fever virus (ASFV; family *Asfarviridae*) in Eastern Europe since 2007 and more recently in European Union (EU) countries has caused considerable concern over the potential for ASFV to spread into Western Europe[1, 2]. ASFV causes a highly virulent disease in domestic pigs with significant economic impact for the pig industry due to a fatality rate of up to 100% and the culling measures and movement bans necessary to control the virus[1, 3]. Containing the ongoing ASF epidemic in Eastern Europe remains a challenge, particularly due to the absence of vaccines or treatment, the stability of the virus in the environment, potential illegal movements of live pigs and pig products, the relatively large number of low-biosecurity farms, the commonplace use of swill feeding, and frequent interactions between domestic and wild swine[4]. In ASF-free European countries, early detection of ASFV introduction is therefore crucial to protect pig health, maintain access to global trade in pigs and pig products, and thereby limit the economic impact of an ASF epidemic. In England, ASF could potentially be introduced through legal and illegal movement of contaminated pig products which may then also subsequently be used for swill feeding. Around 23% of pig farmers interviewed in England have reported using swill to feed their pigs although this practice was banned in the United Kingdom in 2011 [5]. In a recent study, this risk of introduction was considered to be one of the highest in the EU, due to the large number of ports, airports and travellers coming from affected areas [6]. Therefore, the possibility of an ASF epidemic represents a significant economic risk, and preparedness for an ASFV incursion is of high importance to the English pig industry.

In England, the current surveillance system for ASFV relies entirely on the immediate and mandatory notification of any clinically suspect animals by pig farmers to their veterinary surgeon [7], who will then initiate an investigation by local Animal and Plant Health Agency (APHA) veterinary surgeons. Blood and tissue samples are collected for laboratory analysis, and diagnostic results should be available within 24–48 hours. While under investigation, suspect farms are placed under movements’ restrictions in order to reduce the risk of ASFV spread via infected pigs, contaminated vehicles or equipment. In the case of ASFV introduction into the country, the compliance of all farmers with this policy should be able to limit the spread of the virus and thereby mitigate the early spread of the epidemic before control strategies can be implemented.

However, a number of factors may delay reporting by farmers. Firstly, the symptoms of ASF are not specific, particularly in the early clinical stage of the disease. Fever, lethargy and loss of appetite are generally observed among infected domestic pigs, in association with sudden deaths within 2–3 weeks [8, 9]. Other symptoms can include vomiting, diarrhoea and haemorrhages. This is similar to other pig diseases, particularly classical swine fever (CSF), or porcine reproductive and respiratory syndrome (PRRS) which is endemic in England[10, 11]. The disease can also express itself in some domestic pigs in more unusual clinical manifestations, such as with neurological symptoms, and in some cases may even be asymptomatic [12, 13]. Previous surveys have concluded that farmers’ awareness is biased towards diseases which occur at higher prevalence [14], such as PRRS. Since an ASF outbreak has never been confirmed in England, it is unlikely that farmers will have much awareness of the disease, if any. This view is supported by the fact that the Agriculture and Horticulture Development Board (AHDB Pork) recently had to cancel seminars on ASF and porcine epidemic diarrhoea virus due to a lack of interest from the pig industry [15].

Farmers’ awareness of specific clinical signs is essential for early detection of any disease by state authorities [16]. In addition, even if farmers are aware of a particular disease, whether and when they decide to report suspected cases will depend on a variety of factors. A previous study suggested that the main reasons for German, Russian and Bulgarian pig farmers not to immediately report suspected ASF cases included lack of knowledge of reporting procedures, the perceived impact a notification could have on their reputation, expectation that laboratory confirmation would take a long time, and the belief they could handle the outbreak themselves without the involvement of veterinary services [17]. These behavioural and motivational factors have not yet been investigated among English pig farmers.

This study was conducted to investigate pig farmers’ knowledge and behaviour in relation to ASF suspicion and reporting in England, using an online questionnaire survey.

## Materials and Methods

### Data collection

A questionnaire was developed to collect data on pig farmers’ knowledge of ASF clinical disease, and behaviour in relation to ASF suspicion and reporting. The questionnaire contained 19 questions and was estimated to take around 10 minutes to complete. To maximise comparability between respondents, the questions were closed (i.e. questions were in a format that restricts respondents to a range of possible options from which they must choose) or semi-closed (i.e. allowing respondents to express their opinion) [18]. The questionnaire was piloted on one English pig farmer and two interviewers from AHDB Pork and the Royal Veterinary College (RVC) to ensure that questions and pre-defined answers were sufficiently clear and relevant, and it was modified based on their feedback. A short introduction explained the reason for the study, and it was emphasised that answers were anonymous and confidential. The questionnaire was divided into four sections. Section 1collected data on farm characteristics and practices, including herd size, production and housing type, number of animal workers and frequency of pig monitoring. Section 2 collected data on the farmers’ knowledge about ASF epidemiology including recent outbreaks, description of clinical signs, and means of spread. In this section, participants were asked to list three clinical signs typical of ASF out of the following: fever, higher mortality, reduced eating, lethargy, diarrhoea, haemorrhages, vomiting, bloody diarrhoea, coughing, ocular discharge, joint swelling, blood in urine and other signs. Section 3 collected data on the farmers’ behaviour in the context of ASF suspicion, to investigate how they would react if they had clinically suspect animals on their farm and possible reasons for a lack of suspicion. Finally, section 4 collected data on the farmers’ behaviour in relation to ASF reporting, including the reporting timeframe, how they view the risk of making false positive reports, and possible reasons for not reporting. At the end of the questionnaire, farmers were asked to rate how likely their answers represent their actual behaviour on a scale from 1 (not at all) to 10 (very much), and to specify how they found out about the survey.

The target population was all pig farmers within England. A sampling frame of pig farmers was not available as data protection regulations precluded the acquisition of farmers’ contact details. Therefore, a sample size calculation was not carried out and the questionnaire was developed as an online survey using the Surveymonkey software (https://www.surveymonkey.com/), and was advertised through various pig farmers’ organisations, including the National Pig Association (NPA), the British Pig Association (BPA) and AHDB Pork, and posted on the web pages of various professional farming journals, including Pig World [19], The Pig Site [20], Farmers Weekly [21], Vet Record [22] and Farming Life [23]. In addition, pig farmers registered in the Animal Health Extra-Mural Studies database held at the RVC were contacted by phone to encourage them to take part in the survey. Data were collected between December 2014 and April 2015. Scientific and ethical clearance to conduct this study was obtained from the Royal Veterinary College Ethics and Welfare Committee, reference number URN 2014 0122H. The complete questionnaire is available as supplementary material in S1 Fig.

### Data analysis

Descriptive statistics were used for farm characteristics and farmers’ knowledge relating to ASF. Multivariable logistic regression analysis was used to identify factors statistically associated with farmers’ ASF suspicion and reporting behaviour. A binary outcome variable was created to distinguish farmers who “would immediately suspect ASF” if they observed clinical signs of fever, high mortality, reduced eating and lethargy on their farm from those who would “wait for a few days and suspect ASF if the pigs do not improve” or “would not suspect ASF” [8, 9]. Another binary outcome variable was created to distinguish farmers who “would immediately report ASF” if they suspected ASF on their farm from those who “would wait for a few days before reporting” or “would not report ASF”. A total of 28 explanatory variables (binary or ordinal) were considered. First, a univariable analysis was performed for each explanatory with respect to outcome variables. The univariable associations were assessed using chi-squared or Fisher’s exact tests if expected cell counts were less than 5. Collinearity between pairs of explanatory variables was examined using Cohen’s kappa coefficients and considered statistically significant if their absolute value was greater than 0.7 [18]. Explanatory variables with a p-value < 0.2 in the univariable analysis were included in a multivariable logistic regression. Variable selection within the multivariable analysis was based on backward elimination using a p-value < 0.05. Interactions of the explanatory variables were tested and included in the final model if significant (p-value < 0.05). Finally, all remaining variables were tested for confounding and included in the final model if inducing more than 25% changes in the coefficients of the significant variables. The regression coefficients were expressed as odds ratios (OR) with 95% confidence intervals (CI). All statistical analyses were performed using R statistical software (version 3.0.2).

## Results

### Farm characteristics

A total of 121 pig farmers completed the questionnaire but 12 were excluded from the analysis because they did not answer all of the questions. Three of them stopped responding to the questionnaire when asked if they had heard of ASF and nine when asked about their attitudes towards ASF suspicion. Farm characteristics of the remaining 109 pig farmers are summarised in Table 1. The majority of respondents were from Yorkshire and the Humber (20.2%, 22/109), East of England (19.3%, 21/109) and South West England (14.7%, 16/109). Most of the farmers owned more than 1,000 pigs (64.2%, 70/109). The majority of farmers used breed-to-finish production (79.8%, 87/109) with pigs housed indoors (66.1%, 72/109). On about half of the farms, pigs were monitored twice per day (49.5%, 54/109), and 39.5% (43/109) had 3–5 animal workers. In the univariable analyses, none of the farm characteristics were significantly associated with farmers’ ASF suspicion behaviour (Table 2), although two of them (herd size and production type) were significantly associated with ASF reporting behaviour (Table 3).

**Table 1:** Farm characteristics described by questionnaire respondents included in the analysis (n = 109).

Variable	Category	Number	Frequency (%)
Region	North East England	10	9.2
	North West England	6	5.5
	Yorkshire and the Humber	22	20.2
	East Midlands	14	12.8
	West Midlands	6	5.5
	East of England	21	19.3
	South East England	10	9.2
	South West England	16	14.7
	Scotland	2	1.8
	Northern Ireland	2	1.8
Herd size	<10	8	7.4
	10–100	13	11.9
	100–1000	18	16.5
	>1000	70	64.2
Production type	Breeding	12	11.0
	Breed-to-finish	87	79.8
	Wean-to-finish	8	7.4
	Finishing	2	1.8
Housing type	Indoor	72	66.1
	Outdoor	23	21.1
	Both	14	12.8
Animal workers	1–2	24	22.0
	3–5	43	39.5
	6–10	24	22.0
	>11	18	16.5
Pig monitoring	Once per day	22	20.2
	Twice per day	54	49.5
	Three times per day	25	22.9
	More frequently	8	7.4

**Table 2:** Risk factor variables associated (*) with the outcome variable “I would immediately suspect ASF if I observed clinical signs of fever, high mortality, reduced eating and lethargy on my farm” in the univariable analysis (p < 0.2).

Explanatory variable	Category	Outcome variable^a^		p-value^b^
		True (%)	Total number	
**Knowledge of ASF**				
I have heard about the recent ASF outbreaks in other countries	True	18.6	97	0.210
	False	0.0	12	
I know the means of ASF spread	True	25.0	56	0.019*
	False	7.5	53	
I know the clinical signs of ASF	True	33.3	36	0.002*
	False	8.2	73	
**Reasons for not suspecting ASF**				
I am more concerned about other diseases than about ASF	True	9.8	82	0.002*
	False	37.0	27	
There is a low probability of ASF on my farm	True	14.1	85	0.339
	False	25.0	24	
There have been no ASF cases in the UK so far	True	16.8	95	0.999
	False	14.3	14	
I rarely hear about ASF from other farmers or from vets	True	10.3	68	0.047*
	False	26.8	41	
I rarely hear about ASF through the media or journals	True	4.7	43	0.008*
	False	24.2	66	
I am not really certain about the clinical signs of ASF	True	8.0	75	0.001*
	False	35.3	34	

^a^Percentage and total number of respondents answering *True* to the scenario “I would immediately suspect ASF if I observed clinical signs of fever, high mortality, reduced eating and lethargy on my farm”

^b^using Fisher’s exact or chi-squared tests at p < 0.2

**Table 3:** Risk factor variables associated (*) with the outcome variable “I would immediately report ASF if I suspected ASF on my farm” in the univariable analysis (p < 0.2).

Explanatory variable	Category	Outcome variable^a^		p-value^b^
		True (%)	Total number	
**Farm characteristics**				
Herd size	<10	50.0	8	0.081*
	10–1000	54.8	31	
	>1000	74.3	70	
Production type	Breeding	50.0	12	0.014*
	Breed-to-finish	72.4	87	
	Wean-to-finish	25.0	8	
	Finishing	100.0	2	
**Knowledge of ASF**				
I have heard about the recent ASF outbreaks in other countries	True	73.2	97	0.000*
	False	16.7	12	
I know the means of ASF spread	True	75.0	56	0.104*
	False	58.5	53	
I know the clinical signs of ASF	True	75.0	36	0.301
	False	63.0	73	
**Perceptions of ASF false reporting**				
I would still think the report would be useful	True	71.7	99	0.002*
	False	20.0	10	
I would feel ashamed or guilty	True	53.3	15	0.361
	False	69.1	94	
A false report would have a negative effect on my relationship with other farmers, vets or third parties such as abattoirs, hauliers, feed suppliers etc.	True	66.7	36	0.999
	False	67.1	73	
A false report would have negative financial consequences for my farm	True	72.1	43	0.478
	False	63.6	66	
**Reasons for not reporting ASF**				
I would feel ashamed or guilty	True	42.9	7	0.216
	False	68.6	102	
I would prefer to deal with the disease by myself	True	40.0	5	0.329
	False	68.3	104	
Reporting procedure involves too much administrative work/too time consuming	True	33.3	12	0.018*
	False	71.1	97	
Reporting ASF would damage my reputation and relationships with other farmers, vets or third parties	True	65.0	20	0.999
	False	67.4	89	
Reporting ASF would affect my ability to sell pigs in the future	True	71.1	38	0.654
	False	64.8	71	
I would not have any feedback after reporting	True	50.0	8	0.435
	False	68.3	101	
I do not know what will happen after reporting	True	61.5	26	0.663
	False	68.7	83	
Reporting ASF would not help in eradicating ASF from the country	True	33.3	3	0.253
	False	68.6	105	
I do not know the procedure for reporting ASF	True	61.5	26	0.663
	False	68.7	83	

^a^Percentage and total number of respondents answering *True* to the scenario “I would immediately report ASF if I suspected ASF on my farm”

^b^using Fisher’s exact or chi-squared tests at p < 0.2

**Fig 1:**
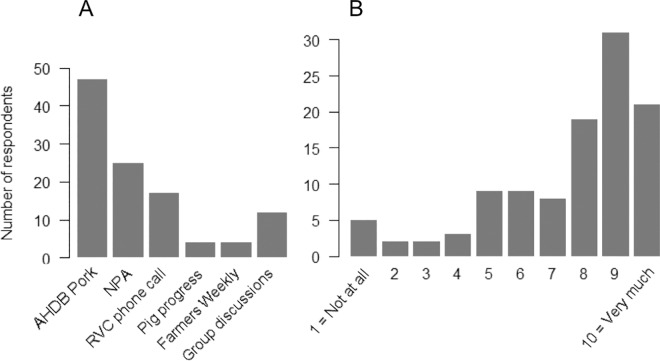
Distribution of A) the means by which respondents found out about the survey and B) farmers’ own rating of the degree that their responses to the questionnaire match their true behaviour in the event of ASF case suspicion.

Most of the participating farmers had heard about the survey through AHDB Pork (43.1%, 47/109) and the NPA (22.9%, 25/109) (Fig 1A). Personal phone calls (15.6%, 17/109) and pig discussion groups (11%, 12/109) encouraged some of the farmers to participate in the survey. There were eight (7.4%) farmers who reported finding out about the survey through pig websites (i.e. Pig Progress and Farmers Weekly). Most farmers (72.5%, 79/109) assigned a score above 7 (on a scale from 1 to 10) to describe the likelihood that their answers would reflect their behaviour if confronted with a possible ASF case (Fig 1B).

### Farmers’ knowledge about ASF clinical signs

**Fig 2:**
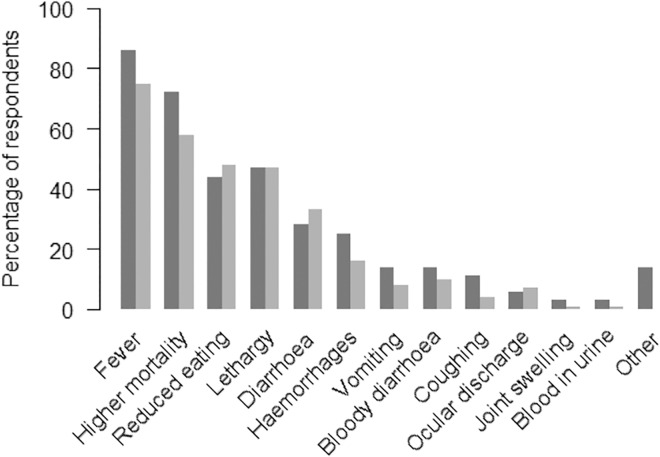
The percentage of indication of clinical signs suggestive of ASF by the farmers who indicated that they did not know the clinical signs (*n* = 36, dark grey) and the farmers who claimed to know the clinical signs (*n* = 73, light grey).

Among the respondents, 89% (97/109) had heard about the recent ASF outbreaks in other countries. There were 51.4% (56/109) and 33.0% (36/109) who answered that they know the means of ASFV spread and ASF clinical signs, respectively. The first three most reported clinical signs suggestive of ASF were fever, higher mortality and reduced eating (Fig 2).

### Factors associated with farmers’ ASF suspicion behaviour

In case of fever, high mortality, reduced eating and lethargy on their farm, most farmers (83.5%, 91/109) would not suspect ASF or would wait for a few days and only suspect ASF if the pigs have not improved by then. However, the majority of farmers (82.6%, 90/109) would immediately seek the opinion of a veterinary surgeon. Six explanatory variables were significantly associated with farmers’ ASF suspicion behaviour in the univariable analysis but no pairwise correlation was identified, resulting in the inclusion of all six variables in the model (Table 2). In the multivariable analysis, three explanatory variables were significantly associated with farmers’ ASF suspicion behaviour (Table 4). Suspicion of ASF was significantly more common among farmers aware of ASF clinical signs (OR = 4.6, 95%CI = 1.5–15.6). Farmers who were less inclined to suspect ASF were more likely to be concerned about other diseases than ASF (OR = 0.3, 95%CI = 0.1–0.9) and uncertain about the clinical signs of ASF (OR = 0.21, 95%CI = 0.0–0.9). No interactions or confounding were found in the final model.

**Table 4:** Risk factor variables statistically significantly associated with the outcome variable “I would immediately suspect ASF if I observed clinical signs of fever, high mortality, reduced eating and lethargy on my farm” in the multivariable analysis (p < 0.05).

Explanatory variable	Category	OR*	95% CI**
I am aware of ASF clinical signs	True	4.6	1.5–15.6
I am more concerned about other diseases than about ASF	True	0.3	0.1–1.0
I am not really certain about the clinical signs of ASF	True	0.2	0.0–0.9

A response of ‘false’ was used as the reference category

*Odds Ratio

**Confidence Interval

### Factors associated with farmers’ suspected ASF case reporting behaviour

Around two thirds of the respondents (67%, 73/109) would quickly report ASF if they suspected it on their farm even if it might be a false positive case. Six explanatory variables were significantly associated with farmers’ reporting behaviour in the univariable analysis but no pairwise collinearity was identified, resulting in the inclusion of all six variables in the model (Table 3). In the multivariable analysis, three explanatory variables were significantly associated with farmers’ reporting behaviour (Table 5). Reporting of ASF was significantly more likely among farmers who were aware of recent ASF outbreaks in other countries (OR = 8.9, 95%CI = 1.8–67.2); those who are not worried about false positive reporting (OR = 10.3, 95%CI = 1.9–80.0); or those who consider that reporting a clinically suspected ASF case does not require too much administrative work and is not too time consuming (OR = 1/0.2 = 5.0, 95%CI = 1.4–33.3). No interactions or confounding were found in the final model.

**Table 5:** Risk factor variables statistically significantly associated with the outcome variable “I would immediately report ASF if I suspected ASF on my farm” in the multivariable analysis (p < 0.05).

Explanatory variable	Category	OR*	95% CI**
I am aware of recent ASF outbreaks in other countries	True	8.9	1.8–67.2
I would still think that reporting cases which proved to be false positives was useful	True	10.3	1.9–80.0
Reporting procedure involves too much administrative work or is time consuming	True	0.2	0.0–0.7

A response of ‘false’ was used as reference category

*Odds Ratio

**Confidence Interval

## Discussion

Results indicate that if noticing ASF-like clinical signs, such as fever, high mortality, reduced eating and lethargy on their farm, most farmers would not suspect ASF and would wait for a few days hoping that the affected pigs’ health improves. This behaviour was statistically significantly associated with farmers’ limited knowledge regarding ASF clinical signs and about the transmission mechanisms. This was also statistically significantly associated with the low importance attributed by farmers to ASF compared with other pig diseases, and the similarity of ASF clinical signs to those of other (including endemic) pig diseases. For example, some respondents pointed out that “PRRS clinical signs are also a fever, lethargy, reduced eating, coughing and wasting”, “I would suspect a PRRS breakdown first” and “There are other diseases which cause similar clinical symptoms”. Despite the fact that most farmers had heard about the outbreaks in other countries, only a third of them were actually aware of what the clinical signs of ASF are. This indicates that disease awareness does not necessarily lead to farmers taking the initiative to obtain better knowledge about the disease. However, it is interesting that there was no difference in knowledge about ASF clinical signs between farmers who indicated having no knowledge about the clinical signs and those who thought they did know. These findings concur with a previous study which emphasised associations between disease prevalence and the level of British pig farmers’ vigilance [24]. Most farmers would consult their veterinary surgeon within a few days, suggesting that the detection of ASF would also rely on veterinary surgeons’ likelihood to suspect ASF on farms as part of a differential diagnosis. Results indicate that a third of farmers would not quickly report ASF if they suspected it on their farm, despite generally positive views in relation to the efficiency of reporting procedures. The likelihood of reporting was significantly higher with increased farmer awareness about outbreaks in other countries. This suggests that while most farmers would support ASF control programs in their compliance with notification procedures, there is still a substantial proportion who would not. Two respondents stated “I don’t know much about reporting ASF” and “I do not know what would happen after reporting a suspicion”.

The results from this study have implications for the design and implementation of ASF surveillance and control strategies. Education and training programmes focusing on the specific knowledge gaps highlighted in this study should be developed and promoted among farmers to improve their ability and willingness to contribute to ASF disease surveillance, to ensure that pigs with ASF-like symptoms are reported to local authorities in a timely manner. Most of the respondents monitored their pigs twice per day, which is sufficiently frequent for effective early disease detection, however, it is likely that farmers will need to perceive a personal benefit if they are to participate in additional education and training. Lack of confidence in learning new skills and low expected impact on farm management practice have previously been highlighted as farmers’ barriers to participating in education and training programmes [25, 26]. Alternatively, small group settings, confidence in speakers respected by the farmers, convenient locations and participatory approaches have been identified as effective delivery methods and as contributing to a greater level of farmer participation [27, 28]. AHDB Pork was recently perceived by English pig farmers as an ‘extremely’ useful source of disease information (through workshops and discussion groups) compared with pig websites [29]. The activities of AHDB Pork are prioritised by pig farmers and other pig industry stakeholders, therefore any investment into ASF education would only occur if the farmers themselves perceived it as being important [30]. Veterinary surgeons and other pig owners should be involved in delivering training programmes as they are perceived by English pig farmers as the most trusted sources of information on disease control [5, 24, 29]. In addition, notification regulations and associated control actions should be clarified (particularly regarding the planned financial compensation policy for pigs culled for ASF control purposes during an outbreak) as this may increase farmers’ likelihood of disease reporting [7].

The results from this survey lead to a number of important conclusions in relation to farmer behaviour in association with ASF suspicion and reporting. However, there are several potentially important biases that need to be taken into account, particularly when attempting to generalise to the whole population of pig farmers in England. Most of the farms included in this survey were located in the regions of Yorkshire and the Humber, the East of England and South West England, which is broadly representative of the spatial distribution of pig holdings in England [31]. According to the latest figures reported by AHDB Pork [32], the English pig industry has a range of production systems, including rearing, grower and finishing farms, and indoor pig production accounts for around 60% of the industry. Respondents mainly had large sized farms (with more than 1000 pigs), which is not representative of the general herd size distribution in England. According to the latest figures reported by DEFRA [33] in 2014, more than 35% of pig farms have less than 10 pigs, and less than 15% have more than 1000 pigs in England. However, the most of pig production industry output comes from a small number of large commercial farms, which are therefore of particular interest for disease surveillance. In addition, large commercial farms account for a high number of pig batch movements to other large commercial farms and markets [31], also highlighting the importance of appropriate disease surveillance at these premises. It is acknowledged that small pig holdings and pet pig owners have also recently been identified as a further target group requiring disease knowledge improvement [5]. The current survey was completed by 109 farmers which represents about 1.5% of all pig holdings in England in 2014 [33]. Various strategies were used to attempt to increase the response rate [34], including keeping the questionnaire short in length, posting a personalised letter with illustrative pictures on websites, and contacting some of the participants before sending the link to the survey. Various pig farmer organisations and professional farming journals also referred to the survey and encouraged farmers to respond, as they recognise ASF is an important topic. It is highly likely that the responding farmers are a biased sample, in that they may be those who perhaps have an interest in ASF and therefore may attribute higher importance to ASF and/or have better knowledge than those who did not respond. Patterns of missing values indicated that there were farmers amongst the respondents who did not wish to discuss their awareness of ASF outbreaks and their behaviour towards ASF suspicion, suggesting that these were either sensitive or irrelevant subjects for them. Respondents were asked to choose three clinical signs from a list that they considered to be related to ASF. It is possible that the choice of clinical signs provided, the number of clinical sign options offered, and the order in which clinical signs were listed has influenced how they responded [18].Among those who did respond to these questions, some farmers may have chosen what they perceived to be the most desirable/expected response, thereby concealing their true behaviour [35], even though interviewees had been assured anonymity and confidentiality of their responses [36].

To date, there have been no confirmed cases of ASF in England. Farmer risk perception and behaviour are likely to change in the event of an outbreak. Firstly, during the course of an outbreak farmers will probably quite quickly improve their knowledge of ASF clinical signs as a result of media exposure. In addition, other factors such as economic aspects (cost of disease and cost-effectiveness of control measures) or livelihood concerns (reputation and relationships with other farmers, vets or third parties) might also emerge as important drivers for ASF suspicion and reporting, as has been suggested in a previous study [29]. In another study, pig farmers expressed their expectation that the British government be responsible for disease knowledge transfer and for the costs of disease controls regarding exotic and notifiable diseases [24]. AHDB Pork, which is a government body, is actively engaged in dissemination of knowledge to pig farmers relating to prevention and control of diseases, although it is funded directly by a statutory levy on pig producers and processors and the allocation of its resources are determined by a board of pig industry stakeholders [30].

Despite the limitations highlighted above, this study provides useful background information on farmer behaviour with respect to the suspicion and reporting of ASF cases in England. It provides data to support the development and optimisation of future farmer education programmes aimed at increasing the likelihood of early detection of ASF outbreaks and therefore a rapid response in the event of ASF incursion into England.

## Supporting Information

S1 Fig Questionnaire survey on pig farmers’ knowledge and behaviour towards African swine fever suspicion and reporting. (PDF)
