# Supplementary material for: English Pig Farmers’ Knowledge and Behaviour towards African Swine Fever Suspicion and Reporting
Source: PLoS One. 2016 Sep 29;11(9):e0161431. doi: 10.1371/journal.pone.0161431 (PMC5042443; doi:10.1371/journal.pone.0161431)
Supplement: S1 Fig — (PDF) [file pone.0161431.s001.pdf]

Introduction

Thank you for taking the time to complete this survey developed by the Royal Veterinary College (RVC) and associated partners (BPEX, NPA, PVS) on the surveillance and control of African swine fever (ASF).

This study is to investigate pig farmers' awareness of ASF and attitudes towards reporting, with the ultimate aim of improving early detection of outbreaks in case of disease incursion in UK.
We are interested in your honest opinions, so that we can develop appropriate mechanisms for early detection of ASF in the UK. Please answer as honestly as possible, there is no 'correct' or 'incorrect' answer.
The questions are mostly checkboxes with "yes/no", "true/false" and "other" answer options.
Anonymised survey results will be available once the data have been analysed.

This survey should not take more than 10 minutes of your time.
Please ensure you complete entirely the survey.
Remember that your answers will be completely anonymous and confidential.

Farm characteristics

*1. In which region of the UK do you live?

Region

*2. How many pigs do you currently have?

- ◯ <10
- ◯ 10-100
- ◯ 100-1000
- ◯ >1000

*3. What is your type of production?

- ☐ Breeding
- ☐ Breed-to-finish
- ☐ Wean-to-finish
- ☐ Finishing
- ☐ Other (please specify)

*4. What type of housing do you have?

- ◯ Indoor
- ◯ Outdoor

Other (please specify)

*5. Do you produce other animal species?

- ◯ Yes
- ◯ No

Other (please specify)

*6. How many animal workers are there on your farm?

- ◯ 1-2
- ◯ 3-5
- ◯ 6-10
- ◯ >11

*7. How often do you monitor your pigs?

- ◯ Once per day
- ◯ Twice per day
- ◯ Three times per day
- ◯ More frequently

African swine fever awareness

*8. Answer the following statements

	Yes	No	Uncertain
Have you ever heard of African swine fever (ASF)?	◯	◯	◯
Have you heard about the recent outbreaks of ASF in other countries?	◯	◯	◯
Do you know the means of spread of ASF?	◯	◯	◯
Do you know the clinical signs for ASF?	◯	◯	◯

*9. Choose 3 clinical signs that you consider mainly related to ASF:

- ☐ Fever
- ☐ Diarrhoea
- ☐ Lethargy
- ☐ Higher mortality
- ☐ Reduced eating
- ☐ Joint swelling
- ☐ Coughing
- ☐ Ocular discharge
- ☐ Vomiting
- ☐ Haemorrhages
- ☐ Bloody diarrhoea
- ☐ Blood in urine
- ☐ Other (please specify)

African swine fever suspicion

*10. If you observed the following clinical signs:
Fever, Lethargy, Reduced eating and No perceptible increase in mortality

	True	False
You would wait a few days to see if the pigs improve or get worse.	◯	◯
You would treat the affected pigs with an antibiotic.	◯	◯
You would immediately seek an opinion from your vet.	◯	◯
You would wait a few days and then seek an opinion from your vet if nothing improves.	◯	◯
You would immediately suspect ASF.	◯	◯
You would wait a few days and suspect ASF if the pigs do not improve or their condition gets worse.	◯	◯

Other (please specify)

*11. If you observed the following clinical signs:
Fever, Lethargy, Reduced eating and High mortality

	True	False
You would wait a few days to see if the pigs improve or get worse.	◯	◯
You would treat the affected pigs with an antibiotic.	◯	◯
You would immediately seek an opinion from your vet.	◯	◯
You would wait a few days and then seek an opinion from your vet if nothing improves.	◯	◯
You would immediately suspect ASF.	◯	◯
You would wait a few days and suspect ASF if the pigs do not improve or their condition gets worse.	◯	◯

Other (please specify)

*12. If you do not suspect ASF, what are the reasons for this?

	True	False
You are more concerned about other diseases than ASF on your farm.	◯	◯
There is a low probability of an ASF outbreak on your farm.	◯	◯
There have been no ASF cases in England so far.	◯	◯
You rarely hear about ASF from other farmers or vets.	◯	◯
You rarely hear about ASF through media or journals.	◯	◯
You are not really certain about the clinical signs of ASF.	◯	◯

Other (please specify)

*13. Have you ever suspected ASF on your farm?

◯ Yes

◯ No

African swine fever reporting

*14. Have you ever reported ASF to your vet or the AHVLA? (Including a false report)

- ◯ Yes
- ◯ No

*15. Within what timeframe would you report ASF if you suspected it on your farm?

	True	False
You would wait a few days before reporting to avoid a false report.	◯	◯
You would wait a few days before reporting due to the financial costs it involves.	◯	◯
You would quickly report ASF, even if it could be a false case.	◯	◯

Other (please specify)

*16. What do you think about reporting ASF if it turns out to be a false report?

	True	False
You would still think the report was useful.	◯	◯
You would feel ashamed or guilty.	◯	◯
This would have a negative image for your farm.	◯	◯
This would have a negative effect on your relationship with other farmers or your vet.	◯	◯
This would have a negative effect on your relationship with third parties such as abattoirs, hauliers, feed suppliers etc.	◯	◯
This would have negative financial consequences for your farm.	◯	◯

Other (please specify)

*17. Why might you not report ASF if you suspected it on your farm?

	True	False
Because you would feel ashamed or guilty.	◯	◯
Because you would prefer to deal with the disease by yourself.	◯	◯
Because it would involve too much administrative work or be too time consuming.	◯	◯
Because it would damage your reputation and relationships with other farmers, vets or third parties.	◯	◯
Because it would affect your ability to sell pigs (either domestic trade or export) in the future. True/False	◯	◯
Because you would not have feedback after reporting.	◯	◯
Because you do not know what will happen after reporting a suspicion.	◯	◯
Because it would not help in eradicating ASF from the country.	◯	◯
Because you do not know the procedure for reporting ASF.	◯	◯

Other (please specify)

These are the two last questions!

***18. To what extent do you feel that your answers represent how you would actually react in the event of African swine fever? 1=not at all to 10=very much**

1	2	3	4	5	6	7	8	9	10
○	○	○	○	○	○	○	○	○	○

***19. How did you hear about this survey?**

- ☐ BPEX
- ☐ NPA
- ☐ PVS
- ☐ Pig progress
- ☐ Farmers Weekly
- ☐ Smallholder
- ☐ Other (please specify)

Conclusion

Thank you very much for having completed the survey!

You will find essential information on ASF suspicion, reporting and prevention at asforce.org through an interactive course focussing on the main aspects that will help you to fight against the disease in case of an outbreak.
